# Biological Control of Crown Gall on Grapevine and Root Colonization by Nonpathogenic *Rhizobium vitis* Strain ARK-1

**DOI:** 10.1264/jsme2.ME13014

**Published:** 2013-05-24

**Authors:** Akira Kawaguchi

**Affiliations:** 1 Research Institute for Agriculture, Okayama Prefectural Technology Center for Agriculture, Forestry and Fisheries, 1174–1 Koudaoki, Akaiwa City, Okayama 709–0801, Japan

**Keywords:** *Rhizobium vitis*, grapevine crown gall, biological control, meta-analysis, root colonization

## Abstract

A nonpathogenic strain of *Rhizobium vitis* ARK-1 was tested as a biological control agent for grapevine crown gall. When grapevine roots were soaked in a cell suspension of strain ARK-1 before planting in the field, the number of plants with tumors was reduced. The results from seven field trials from 2009 to 2012 were combined in a meta-analysis. The integrated relative risk after treatment with ARK-1 was 0.15 (95% confidence interval: 0.07–0.29, *P*<0.001), indicating that the disease incidence was significantly reduced by ARK-1. In addition, the results from four field trials from 2007 to 2009 using *R. vitis* VAR03-1, a previously reported biological control agent for grapevine crown gall, were combined in a meta-analysis. The integrated relative risk after treatment with VAR03-1 was 0.24 (95% confidence interval: 0.11–0.53, *P*<0.001), indicating the superiority of ARK-1 in inhibiting grapevine crown gall over VAR03-1 under field conditions. ARK-1 did not cause necrosis on grapevine shoot explants. ARK-1 established populations on roots of grapevine tree rootstock and persisted inside roots for two years.

Grapevine crown gall, caused mainly by *Rhizobium vitis* (Ti; “Ti” means “tumor-inducing” or “tumorigenic”) (= *Agrobacterium vitis* [Ti], *A. tumefaciens* biovar 3), is the most important bacterial soil-borne disease of grapevines in the world (4, 5, 22). There is no effective control method at present. The pathogenicity genes are mostly located on large tumor-inducing plasmids (pTi). During infection, a part of this plasmid (T-DNA) is transferred and inserted into the nuclear DNA of the plant (7).

Several laboratories have attempted to identify biological measures to control grapevine crown gall (3, 5, 6, 8, 23, 24, 25). Staphorst *et al.* (23) evaluated nonpathogenic *R. vitis* strain F2/5, which inhibited the growth of most tumor-inducing strains of *R. vitis* in vitro and greatly inhibited crown gall on grapevine in stem-wounding experiments in greenhouse experiment. Burr and Reid (5) reported that F2/5 produces agrocin, which inhibits most *R. vitis* (Ti) strains in vitro, and effectively inhibits tumor formation at wound sites on grapevine stems artificially inoculated with one of several *R. vitis* (Ti) strains; however, F2/5 did not inhibit tumor formation caused by other strains of *R. vitis* (Ti) (4), and F2/5 caused necrosis on grapevine shoot explants (9).

Previously, the author reported that a nonpathogenic *R. vitis* strain, VAR03-1, isolated from nursery stock of grapevine in Japan, greatly inhibited tumor formation on grapevine (13–15). Moreover, nonpathogenic *R. vitis* strain ARK-1, which was better at inhibiting tumor formation on grapevine than VAR03-1, was identified as a new antagonistic strain (12). ARK-1 did not produce a halo of inhibition around *R. vitis* (Ti) strain on yeast-mannitol agar (YMA) medium, and ARK-1 did not reduce tumor incidence on the stems of grapevine when ARK-1 was killed by autoclave or only the culture filtrate was used, indicating that ARK-1 inhibits grapevine crown gall in planta by a different mechanism than VAR03-1 (12). The final purpose of this study was to utilize strain ARK-1 as a biopesticide; however, there is no evidence of the effectiveness of treatment with strain ARK-1 in controlling grapevine crown gall in the field.

This article reports that strain ARK-1 reduced the frequency of grapevine crown gall in 7 field trials and colonized on grapevine roots for 2 years. Moreover, the effectiveness of ARK-1 and VAR03-1 under field conditions was compared in this article. The report follows the nomenclature for *Rhizobium* species adopted in the reports of Bull *et al.* (2) and Young *et al.* (26) to avoid confusion, although other valid naming systems have been proposed (1, 18–20, 27).

## Materials and Methods

### Biological control in field trial

Eleven trials (2006-A, 2007-A, 2007-B, 2009-A, 2009-B, 2009-C, 2010-A, 2010-B, 2011-A, 2011-B, and 2012-C) designed as randomized or systematic controlled trials of biological control of grapevine crown gall were carried out in three different experimental fields, A (2006, 2007, 2009, 2010, and 2011), B (2009, 2010, and 2011), and C (2007 and 2012), of the Okayama Prefectural Technology Center in Akaiwa City, Okayama, Japan. Trials 2007-A and 2007-B were previously reported (15). The sizes of experimental fields A, B, and C are 144.0 m^2^ (24.0 m×6.0 m), 28.8 m^2^ (9.0 m×3.2 m), and 45.0 m^2^ (15.0 m×3.0 m), respectively. All field trials except 2007-B were carried out using grapevine nursery stock (scion cultivar: *Vitis vinifera* × *V. labrusca* cv. Pione, rootstock: *V. cinerea* var. *helleri* × *V. riparia* cv. Teleki-Kober 5BB) grown from cuttings (2 years old). Trial 2007-B was carried out using small grapevine seedlings (*V. vinifera* cv. Neo Muscat, 1 year old). One month before the trials, a commercial organic fertilizer (Temporon, containing N=0.77%, P=0.09%, K=0.08%, lignocellulose, humic acid, Ca, Mg, Mn, and B; Mitsubishi-Shoji, Tokyo, Japan) was applied at a rate of 4.0 kg m^−2^ and thoroughly incorporated into the soil of the fields every year. *R. vitis* (Ti) strains were divided into five genotypes (A to E) (11, 17). Seven typical strains of *R. vitis* (Ti) belonging to genotypes A to E isolated in Japan were selected as the pathogen (Table 1). Two weeks before the trials, 20 L m^−2^ of a mixed cell suspension (about 10^8^ cells mL^−1^) of *R. vitis* (Ti) strains G-Ag-27 (Genotype A), MAFF211676 (Genotype A), MAFF211674 (Genotype B), G-Ag-60 (Genotype C), VAT07-1 (Genotype C), UK-2 (Genotype D), and IS552-1 (Genotype E) was poured onto the soil, and then soil was broken up to a depth of 16 cm by a Punch-X F402-J cultivator (Honda Motor, Tokyo, Japan) to disperse the inoculum in the soil every year. The cell suspension, which was a mixture of the seven tumorigenic strains, was prepared from 48-h liquid cultures grown on potato semi-synthetic (PS) medium (300 g potato extract, 0.5 g Ca (NO_3_)_2_·4H_2_O, 2 g Na_2_HPO_4_·12H_2_O, 5 g peptone, 20 g sucrose, and 1 L distilled water, pH 6.8–7.0). In trials 2006-A, 2007-A, and 2007-B, a mixed cell suspension (about 10^8^ cells mL^−1^) of *R. vitis* (Ti) strains At-90-23 (Genotype A), G-Ag-27, MAFF211676, and MAFF211674 was used.

The condition of each field trial is shown in Table 2. Cell suspensions of strains ARK-1 and VAR03-1 were prepared from 48-h slant cultures grown on PS agar (PSA) medium (PS medium with 15 g agar) and adjusted to OD_600_=0.2 (corresponding to about 2×10^8^ cells mL^−1^) and 1.0 (corresponding to about 1×10^9^ cells mL^−1^), respectively. Roots of plants were pruned to half and soaked for 1 h in a cell suspension of strain ARK-1, VAR03-1, or water, and then 16 to 45 plants per treatment were planted in each plot. The arrangement of each plot was random or systematic within each field. Tumor formation on roots and stems of plants was investigated after six to nine months. The rainy season in Okayama, Japan was from June to July. The temperature ranged from 10°C to 37°C, and no severe damage by weather or insects was observed during cultivation.

The disease incidences in the seven field trials of ARK-1 treatment and four field trials of VAR03-1 treatment were subjected to meta-analysis according to a random effect model by the DerSimonian-Laird method (21) because the field trials were performed in different plots sizes, numbers of plots, and plants, farms, and years. Meta-analysis is a set of statistical procedures for synthesizing research results from a number of different studies (21). The DerSimonian-Laird method can incorporate variations among studies (21). An estimate of the statistical effect, such as the difference in disease severity for plants with or without treatment, is collected from each study along with a measure of the variance of the estimate of the effect. The effect size of antagonist treatment was calculated as integrated relative risk. Relative risk was defined as Relative risk=(proportion of plants with tumors in antagonist treatment)/(proportion of plants with tumors in water treatment). Meta-analyses were performed using EZR (10), which is a graphical user interface for R (The R Foundation for Statistical Computing, version 2.14.0). The tumor formation ratio was defined as Tumor formation ratio=100×(total number of tumors in antagonist treatment)/(total number of tumors in water treatment).

### Necrosis assay

The necrosis assay based on previous reports of Herlache *et al.* (9) was carried out using grapevine cv. Pione green shoot explants. Explants were excised from greenhouse-grown vines and surface-disinfected with a 50% (vol/vol) solution of bleach in distilled water for 20 min followed by 70% ethanol for 5 min. They were rinsed in sterile distilled water and cut into approximately 1.0 to 1.5 cm long sections. Explants were supported vertically, with their basal end up, in 4% water agar plates, and their aerial ends were inoculated with 2 μL drop of ARK-1 (about 10^9^ cells mL^−1^) or sterile distilled water as a negative control. Thirty explants were inoculated with ARK-1 and sterile distilled water in each experiment. The experiment was repeated three times. Development of necrosis was assessed over a period of 5 d.

### Population dynamics of strain ARK-1 on grapevine root

In the survival assay of grapevine roots, antibiotic-resistant mutants of ARK-1sc were used to differentiate inoculated biological control agents from indigenous agrobacteria. ARK-1sc was a streptomycin (St) -copper sulfate (CuSO_4_)-resistant mutant (St-CuSO_4_-mutant) obtained by growing strain ARK-1 on St-CuSO_4_-PSA medium (amended with 500 ppm St and 250 ppm CuSO_4_) (16). The survival rate of nonpathogenic strain ARK-1sc in the grapevine root was determined. Eight nursery stocks of grapevine (scion cultivar: cv. Pione, rootstock: cv. Teleki-Kober 5BB) grown from cuttings (2 years old) were prepared. A cell suspension of ARK-1sc contained 2×10^8^ cells mL^−1^. Roots of 8 plants per treatment were pruned into half, soaked for 1 h in a cell suspension of strains ARK-1sc and planted in concrete-frame plots (1.0 m×1.0 m plot^−1^, 1.0 m tall, in the field) filled with soil (pH=5.8, NO_3_-N=7.9 mg 100 g^−1^ soil, P_2_O_5_=131 mg 100 g^−1^ soil, K_2_O=32 mg 100 g^−1^ soil, CaO=281 mg 100 g^−1^ soil, cation exchange capacity=26.2 meq 100 g^−1^ soil, organic matter content=1.8%) on 19 October 2010. Plants not treated with the test strain were prepared as a negative control. To determine the populations of ARK-1sc, roots (1 g fresh weight per plant) were collected from 8 plants. Each piece was scrubbed by hand, rinsed under tap water for 10 s and dried with paper towels. To wash the surface of roots, each piece was incubated with 1 mL sterile distilled water at 20°C with shaking (approximately 200 rpm) for 1 h. In order to isolate ARK-1sc from the root surface, serial dilutions of the water collected after incubation were plated on St-CuSO_4_-PSA supplemented with 500 ppm tebuconazole (Bayer Cropscience, Tokyo, Japan) to avoid the influence of contamination by fungi on the roots. In order to isolate ARK-1sc from inside the roots, the root collected after incubation was mashed with an autoclaved mortar and pestle in 1 mL sterile distilled water, and then serial dilutions of the samples were plated on St-CuSO_4_-PSA supplemented with 500 ppm tebuconazole. The plates were incubated at 27°C for 5 d. The observations were based on 10 plates of each dilution, and the number of colony forming units (CFU) of strain ARK-1sc was counted on each medium. The bacterial population on the root was transformed as a logarithm (base 10) of CFUs per gram of root for analysis.

## Results and Discussion

**Fig. 1:**
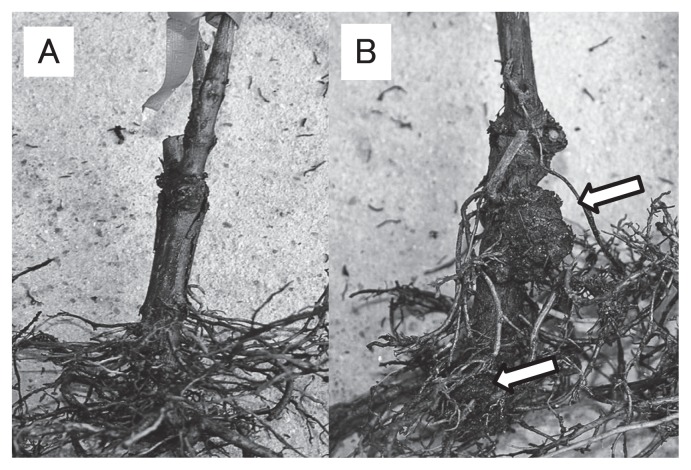
Biological control of grapevine crown gall by nonpathogenic *Rhizobium vitis* strain ARK-1. Treatment with a cell suspension of strain ARK-1 (A). Treatment with water (B). Tumors (*arrow*) developed on the roots and stems. The photograph was taken approximately 6 months after treatment in 2009-A field trial.

**Fig. 2:**
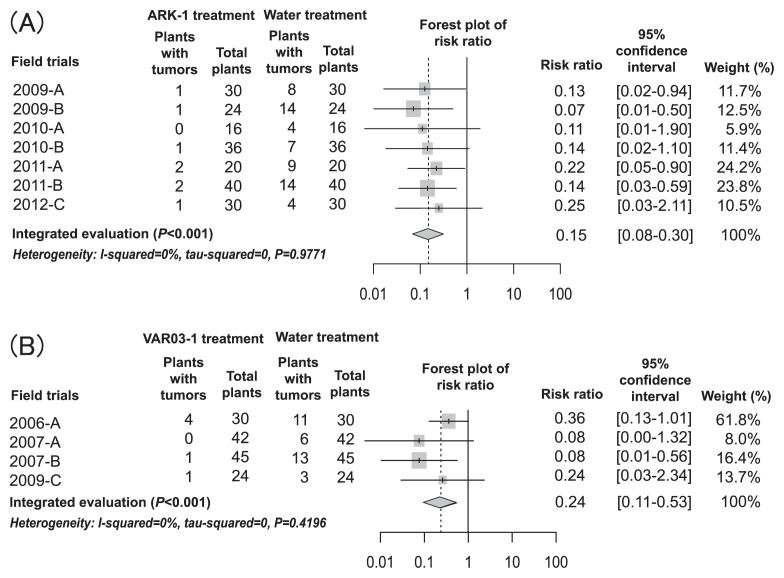
Integrated evaluation based on meta-analysis of the effect of nonpathogenic *Rhizobium vitis* strains ARK-1 (A) and VAR03-1 (B) on grapevine crown gall after soaking plant roots in bacterial cell suspensions in field trials. The center and width of the diamond shape demonstrates the value of the integrated risk ratio and 95% confidence interval, respectively.

As shown in Fig. 1, tumor formation on roots and stems of plants was investigated. The meta-analysis results from the seven field trials performed from 2009 to 2011 regarding the biological control effect of strain ARK-1 on grapevine crown gall are shown (Fig. 2A). The integrated relative risk was 0.15 (95% confidence interval: 0.07–0.29, *P*<0.001), indicating that the disease incidence was significantly reduced by ARK-1 (Fig. 2A). The integrated relative risk value 0.15 indicates that the incidence of crown gall disease during treatment with ARK-1 decreased to 15% of that without ARK-1 and that the control effect was very high in the field. Thus, the integrated relative risk value 0.15 makes ARK-1 very useful in the field. There are no reports of a biological control agent that is better at inhibiting tumor formation on grapevine in the field than ARK-1. In addition, the meta-analysis results from the four field trials performed from 2006 to 2009 regarding the biological control effect of strain VAR03-1 are shown (Fig. 2B). The integrated relative risk was 0.24 (95% confidence interval: 0.11–0.53, *P*<0.001), indicating that the crown gall disease incidence during treatment with VAR03-1 had decreased to 24% of that without VAR03-1 (Fig. 2B). Although these two meta-analyses were performed using different numbers of field trials and were not performed at the same time in the same field, the results of field trials comparing the effectiveness of ARK-1 and VAR03-1 indicate the superiority of ARK-1 over VAR03-1; however, the integrated relative risk value 0.24 is highly effective for control and is useful in the field.

The heterogeneity between each study was tested in each meta-analysis of the inhibitory effects of ARK-1 and VAR03-1 treatments. The I-squared value of each was 0%, and *P* values were 0.9771 (seven field trials of ARK-1 treatment) and 0.4196 (four field trials of VAR03-1 treatment), indicating no heterogeneity among field trials.

Meta-analysis of the seven field trials showed strong evidence that ARK-1 was effective in controlling grapevine crown gall by application in the field, indicating that soaking for one hour in a cell suspension of 2×10^8^ cells mL ARK-1 is suitable for practical use. To develop a new bactericide with ARK-1, it is necessary to investigate whether it is effective with a lower density of cell suspension than used this study.

The number of tumors that developed on grapevine was reduced by ARK-1 and VAR03-1 because the means of the tumor formation rates of ARK-1 and VAR03-1 treatments were 15% and 19%, respectively (Table 3), indicating that ARK-1 and VAR03-1 could reduce disease severity.

A necrosis assay of ARK-1 was carried out using grapevine cv. Pione green shoot explants. In three experiments, there was no necrosis in ARK-1- and sterile distilled water-treated plants after 5 d. Necrosis of grapevine tissues may also be caused by certain *R. vitis* strains (9). F2/5 caused necrosis on grapevine shoot explants within 72 h after inoculation (9). The results of the present study indicate that ARK-1 was a different type of antagonistic strain from F2/5. Moreover, we observed no necrosis on grapevine roots inoculated with ARK-1 in these biological control trials.

**Fig. 3:**
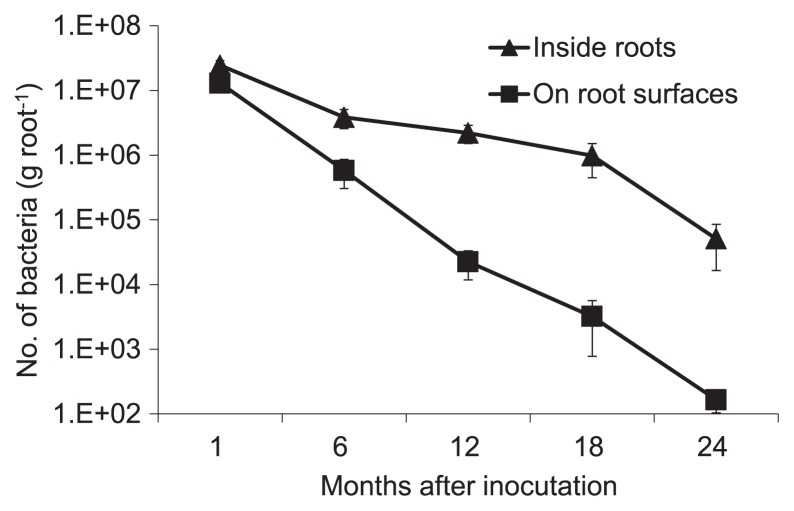
Population dynamics of nonpathogenic *Rhizobium vitis* strain ARK-1sc in the roots of grapevine after inoculation. Data are the means of eight rootstocks. Error bars represent the standard error of the mean.

As shown in Fig. 3, six months after inoculation with strain ARK-1sc, the bacterial population inside roots was 4×10^6^ CFU g^−1^ (fresh weight) of root. Colonization by ARK-1sc inside roots remained at 2×10^6^ CFU g^−1^ of root for up to 12 months, and then dropped to 5×10^4^ CFU g^−1^ of root after 24 months. On the other hand, six months after inoculation with strain ARK-1sc, the bacterial population on root surfaces was 6×10^5^ CFU g^−1^ of root. Colonization by ARK-1sc on root surfaces remained at 2×10^4^ CFU g^−1^ of root for up to 12 months, and then dropped to 2×10^2^ CFU g^−1^ of root after 24 months. Previously, the authors reported that colonization of grapevine roots by VAR03-1 remained at about 10^6^ CFU g^−1^ of root for up to 1 year, and then dropped to about 10^4^ CFU g^−1^ of root after 2 years (15). In the survival assay on the roots of grapevine seedlings, the result for ARK-1sc indicated that strain ARK-1 not only established populations in the rhizosphere of grapevine but also persisted inside roots for up to 2 years. This result suggested that the bacterial population treated with strain ARK-1 was almost the same as that treated with VAR03-1 for up to 2 years. On the other hand, colonization of ARK-1sc on root surfaces remained at 2×10^4^ CFU g^−1^ of root for up to 12 months, and then dropped to 2×10^2^ CFU g^−1^ of root after 24 months. These comparisons of the survival of ARK-1sc inside roots and on root surfaces demonstrated that the bacterial population on root surfaces was always lower than that inside roots, indicating the possibility that ARK-1 is an endophytic bacterium. Incidentally, strain ARK-1 was isolated from grapevine tissue (12). We plan to continue investigating the bacterial population treated with strain ARK-1sc until ARK-1sc cannot be isolated from the roots of grapevines treated in this study. The ability to colonize roots might affect the persistence of the control of grapevine crown gall. Thus, the persistence of the control of grapevine crown gall by ARK-1 should be investigated in detail.

## Conclusions

This is the first study to report that a nonpathogenic *R. vitis* strain, ARK-1, effectively controlled grapevine crown gall in field trials. The result of field trials comparing the effectiveness of ARK-1 and VAR03-1 indicated the superiority of ARK-1 to VAR03-1. Further, this study showed that ARK-1 not only established populations in the rhizosphere of grapevine but also persisted inside roots for up to two years. The applicability of ARK-1 to other kinds of plants in the field should be investigated further.

## Figures and Tables

**Table 1:** Bacterial strains used in this study

Bacterial strain^a^	Pathogenicity^b^	Genotype of *R. vitis*^c^	Opine type	Description (supplier)
*Rhizobium vitis* (Ti) (= *Agrobacterium vitis* [Ti], *A. tumefaciens* biovar 3)				
At-90-23	Ti	A	Unknown	Isolated by J. Yamamoto from galled grapevine trees in Japan (J. Yamamoto) (15)
G-Ag-27	Ti	A	Vitopine	Isolated by H. Sawada from galled grapevine trees in Japan (H. Sawada) (12)
MAFF211676	Ti	B	Unknown	Isolated by A. Kawaguchi from galled grapevine trees in Japan (12)
MAFF211674	Ti	A	Unknown	Isolated by A. Kawaguchi from galled grapevine trees in Japan (12)
G-Ag-60	Ti	C	Nopaline	Isolated by H. Sawada from galled grapevine trees in Japan (H. Sawada) (12)
VAT07-1	Ti	C	Nopaline	Isolated by A. Kawaguchi from galled grapevine trees in Japan (12)
UK-2	Ti	D	Octopine	Isolated by T. Misawa from galled apple trees in Japan (T. Misawa) (12)
IS552-1	Ti	E	Unknown	Isolated by T. Misawa from galled apple trees in Japan (T. Misawa) (12)
Nonpathogenic *R. vitis* (=Nonpathogenic *A. vitis*, *A. radiobacter* biovar 3)				
ARK-1	N	F	…	Isolated by A. Kawaguchi from nursery stock of grapevine in Japan; biological control agent for crown gall (12)
VAR03-1	N	F	…	Isolated by A. Kawaguchi from nursery stock of grapevine in Japan; biological control agent for crown gall (13, 14, 15, 16)
ARK-1sc	N	…	…	Streptomycin- and copper sulfate-resistant mutant of strain ARK-1 (12)

a MAFF: Ministry of Agriculture, Forestry and Fisheries, Tsukuba, Ibaraki, Japan.

b Ti: Tumorigenic; N: Nonpathogenic.

c Genetic group based on multilocus sequense analysis of housekeeping genes *pyrG*, *recA*, and *rpoD* (11, 17).

**Table 2:** Conditions of 11 field trials

Trial	Antagonist	Plot size (m)	Plot arrangement	Total no. of plants/treatment	No. of plots/treatment	No. of plants/plot	No. of rows/plot^b^	Date planted/investigated
2006-A	VAR03-1	8.0×3.0	Systematic	30	2	15	3 rows spaced 50cm apart and 100 cm between plants	28-Mar./28-Sep.
2007-A^a^	VAR03-1	8.0×3.0	Randamized	42	3	14	2 rows spaced 60 cm apart and 40 cm between plants	19-Apr./27-Nov.
2007-B^a^	VAR03-1	1.6×1.5	Randamized	45	3	15	6 rows spaced 15 cm apart and 15 cm between plants	13-Feb./12-Oct.
2009-C	VAR03-1	7.0×1.0	Randamized	24	3	8	1 row spaced 50 cm between plants	21-Apr./4-Nov.
2009-A	ARK-1	6.0×1.0	Randamized	30	3	10	1 row spaced 50 cm between plants	11-May/4-Nov.
2009-B	ARK-1	1.6×1.5	Randamized	24	6	4	1 row spaced 40 cm between plants	25-Apr./9-Jan.
2010-A	ARK-1	6.0×1.0	Systematic	16	2	8	1 row spaced 40 cm between plants	26-May/5-Oct.
2010-B	ARK-1	1.6×1.5	Randamized	36	6	6	2 rows spaced 60 cm apart and 40 cm between plants	10-Mar./18-Oct.
2011-A	ARK-1	6.0×1.0	Systematic	20	2	10	1 row spaced 50 cm between plants	28-Mar./5-Dec.
2011-B	ARK-1	1.6×1.5	Randamized	40	4	10	2 rows spaced 60 cm apart and 30 cm between plants	24-Mar./21-Dec.
2012-C	ARK-1	7.0×1.0	Randamized	30	3	10	1 row spaced 60 cm between plants	5-Apr./6-Nov.

a See previous report (15).

**Table 3:** Effect of nonpathogenic *Rhizobium vitis* strains ARK-1 and VAR03-1 on grapevine crown gall after soaking plant roots in bacterial cell suspensions in 11 field trials

Trial	Antagonist	Treatment with antagonist						Treatment with water			
	
		No. of plants with tumors	No. of healthy plants	Plants with tumors (%)	Total no. of tumors^b^	Tumor formation ratio (%)^c^	Mean of tumor formation ratio (%)	No. of plants with tumors	No. of healthy plants	Plants with tumors (%)	Total no. of tumors^b^
2006-A	VAR03-1	4	26	13	6	35		11	19	37	17
2007-A^a^	VAR03-1	0	42	0	0	0		5	37	12	5
2007-B^a^	VAR03-1	1	29	3	1	6		13	32	29	18
2009-C	VAR03-1	1	23	4	2	33	19	4	20	17	6

2009-A	ARK-1	1	29	3	1	7		8	22	27	14
2009-B	ARK-1	1	23	4	1	6		14	10	58	18
2010-A	ARK-1	0	16	0	0	0		4	12	25	4
2010-B	ARK-1	1	35	3	1	14		7	29	19	7
2011-A	ARK-1	2	18	10	3	33		9	11	45	9
2011-B	ARK-1	2	38	5	3	20		14	26	35	15
2012-C	ARK-1	1	29	3	1	25	15	4	26	13	4

a See previous report (15).

b This number is the total tumors formed in each plant.

c Tumor formation raito (%) =100×(total no. of tumors in treatment with antagonist)/(total no. of tumors in treatment with water).
